# The Outcomes of Cochlear Implantation in Usher Syndrome: A Systematic Review

**DOI:** 10.3390/jcm10132915

**Published:** 2021-06-29

**Authors:** Camron Davies, Jenna Bergman, Carly Misztal, Renuka Ramchandran, Jeenu Mittal, Erdogan Bulut, Viraj Shah, Rahul Mittal, Adrien A. Eshraghi

**Affiliations:** 1 Cochlear Implant and Hearing Research Laboratory, Department of Otolaryngology, University of Miami Miller School of Medicine, Miami, FL 33136, USA; cdavi191@med.fiu.edu (C.D.); jberg062@med.fiu.edu (J.B.); cmisztal@med.miami.edu (C.M.); r.ramchandran@med.miami.edu (R.R.); j.mittal@med.miami.edu (J.M.); erdoganbulut@gmail.com (E.B.); viraj.shah@med.miami.edu (V.S.); r.mittal11@med.miami.edu (R.M.); 2 Department of Neurological Surgery, University of Miami Miller School of Medicine, Miami, FL 33136, USA; 3 Department of Biomedical Engineering, University of Miami, Coral Gables, FL 33146, USA; 4 Department of Pediatrics, University of Miami Miller School of Medicine, Miami, FL 33136, USA

**Keywords:** Usher Syndrome, cochlear implant, systematic review, speech perception, audiologic outcomes

## Abstract

Objective: To systematically appraise the implementation of cochlear implantation (CI) in Usher Syndrome (USH) Types 1, 2, and 3 patients, and analyze who would benefit from CI. Data Sources: A comprehensive search of PubMed, Embase, CINAHL, and Cochrane Library electronic databases from inception through June 2020 was performed. There were no language restrictions. Study Selection: The PRISMA strategy was followed. Included studies discuss USH patients who underwent CI regardless of age, nationality, or clinical subtype. All included studies report post-implantation functional, cognitive, or quality of life outcomes. Only reviews were excluded. Results: Fifteen studies met the inclusion criteria. USH patients experienced improvements in PTA and speech perception and expression outcomes after CI, as well as improvements in phonological memory and quality of life measures. Overall, patients implanted at younger ages outperformed older patients in audiological testing. Similarly, patients with prolonged auditory deprivation had relatively poor performance outcomes in sentence recognition and speech detection following CI. Conclusions: Most USH patients benefit from CI. USH patients who undergo CI at younger ages generally achieve better hearing, speech, and cognitive outcomes. CI at older ages can still prove beneficial if appropriate auditory amplification is started at the right time. Further research is warranted to fill the gap in understanding regarding the gene mutations underlying the pathophysiology of USH that have favorable CI outcomes as well as the optimal time to perform CI.

## 1. Introduction

### 1.1. Clinical Presentation

Usher Syndrome (USH) is a rare autosomal recessive disorder characterized by sensorineural hearing loss, retinitis pigmentosa, and varying levels of vestibular function [1]. Three distinct clinical subtypes of USH (Types 1, 2, and 3) have been described and are distinguished based on audiovestibular findings [2]. Clinical subtype characteristics are summarized in Table 1.

#### 1.1.1. USH1

USH1 is the most severe subtype, characterized by congenital profound bilateral hearing loss, early-onset retinitis pigmentosa, and vestibular areflexia [3,4]. Seven genetic subtypes (“A” through “G”) have been implicated in the pathogenesis of USH1, and each of the identified mutations involve proteins that are vital to the function of inner ear mechanoreceptors [5]. Residual hearing is only detectable at low frequencies, and night blindness usually develops before puberty [6]. Early hearing intervention for auditory rehabilitation is necessary to prevent the devastating dual handicap which occurs with the progressive deterioration of visual acuity. The clinical outcomes of cochlear implants in this population are highly dependent on the age of implantation, duration of deafness, and preoperative modes of communication [7].

#### 1.1.2. USH2

USH2 presents as congenital moderate to severe bilateral hearing loss, retinal degeneration starting as early as adolescence, and normal vestibular function. Four genetic subtypes of USH2 have been described; however, there is no clear correlation between these mutations and their respective auditory phenotypes [5]. The hearing loss in USH2 is associated with a high-frequency sloping configuration. It has been shown to rapidly progress with an average annual threshold increase of 0.7 dB per year [5,8]. It is challenging to differentiate USH2 from USH1 since both can lead to hearing and visual impairment very early in childhood; however, the critical distinguishing feature between these two subtypes is the preserved vestibular function seen in USH2. 

#### 1.1.3. USH3

USH3 patients have progressive hearing loss with variable age of onset, progressive vestibular dysfunction, and often a later onset of retinal degeneration compared to the other subtypes [8,9]. USH3 also has a highly unpredictable presentation regarding the type and extent of hearing loss. This is reflected in the hearing tests, which range from normal to moderate hearing impairment similar to USH2 to the severe impairment seen in USH1. Moreover, unique among the usher syndromes is that the degree of impairment increases with age at variable rates, with some patients barely progressing and others going from almost normal hearing to almost complete deafness. A Finnish study used serial pure tone audiometry (PTA) in 59 USH3 patients taken between 1–40 years follow-up interval with an average age of 47 years who used hearing aids before cochlear implantation. They found the progression follows a nonlinear path, slowing with age, and is most apparent within the first 20 years of life [10]. On a cellular level, while the precise function of the USH3A protein in cochlear hair cells and spiral ganglion cells is unknown; the clinical variability is thought to be due to multiple splice variants of the USH3A protein resulting in varying functionality [11]. This highly variable presentation presents particularly unique challenges for the early use of CI in USH3 patients. While early CI leads to better performance outcomes, CI in patients with near-normal hearing could present an unnecessary risk to their residual hearing and burdensome lifetime expense.

In patients with suspected USH, the initial clinical picture may not always be the same. Aside from hearing loss that is detected with universal newborn screening, USH1 typically presents as a delay in gross motor milestones due to vestibular dysfunction, with more severe gait abnormalities occurring as vision loss progresses [4]. Meanwhile, motor milestones are generally normal in USH3, as symptoms often manifest in later childhood [5,12]. The presenting symptoms of retinopathy in all types of USH are night vision loss and visual field restriction, followed by decreasing visual acuity that eventually leads to blindness [12].

### 1.2. Etiology and Epidemiology

USH is characterized by genetic heterogeneity and is inherited in an autosomal recessive manner. Thirteen genes and sixteen mapped chromosomal loci have been implicated in USH, with different genes associated with each clinical subtype [13]. These genes code for proteins that are hypothesized to exist as an “interactome” in the inner ear and retina. In the inner ear, these proteins are co-localized to the stereocilia and hair cell synaptic regions, with USH mutations affecting mechanoelectrical signal transduction [5].

The prevalence of USH ranges from 1/6000 to 1/10,000 [14]. In the typical population, USH occurs in 4 to 17 of every 100,000 people and is found in 3–6% of children with congenital deafness [15,16]. The prevalence of USH also differs depending on the clinical subtype, with USH1 and USH2 accounting for the greatest proportion of cases. USH3 is rare, accounting for about 2% of USH cases overall but up to 40% of cases in Finnish and Ashkenazi Jewish populations [8]. 

### 1.3. Diagnosis

The diagnosis of USH is traditionally made on a clinical basis. Universal newborn hearing screening allows for the earlier diagnosis of USH, specifically in clinical subtypes that present with congenital hearing loss [17]. Further workup for an abnormal newborn screening includes otoscope visualization for otitis media, cytomegalovirus testing, temporal bone imaging, and genetic testing if syndromic hearing loss is suspected [18]. Vestibular involvement can be assessed with caloric testing, rotary chair testing, electronystagmography, and posturography [17,19]. A thorough ophthalmologic examination is also warranted in suspected USH patients, as audiologic manifestations of USH often precede visual symptoms. Workup includes frequent retinal visualization, electroretinogram, and optical coherence tomography to monitor progressive changes [17].

A clinical diagnosis of USH can be confirmed with genetic testing for known pathogenic variants. Next-Generation Sequencing multi-gene panels are used to detect coding-exon mutations in implicated genes. Sanger sequencing can be used in cases of low coverage or family studies [20]. Patients with a confirmed genetic diagnosis can be further characterized based on audiological parameters. It has been demonstrated that a simple audiological evaluation could distinguish between patients under forty years old with USH type IB and type 2A at low frequency (0.25–1k Hz) [21]. Additionally, the genetic mutation profile of USH patients may vary between populations, so mutation analysis must be tailored to the patient based on his or her background [22,23,24]. The full scope of genes implicated in USH is still being uncovered despite advancements in molecular analysis.

### 1.4. Current Use of Cochlear Implantation

Moreover, despite being an overall cost-effective treatment, CI has an estimated lifetime cost of over $1,000,000 [25,26]. Therefore, it has been suggested to minimize the use of CI in patients who would not fully benefit from the procedure.

Bilateral CI as it is thought to have superior results compared to unilateral implantation and has also shown improvements in sound localization. This is due to enhanced brain plasticity, binaural squelch effect, and the head shadow effect [27]. This hypothesis was supported by the LOCHI study, which found that early functional hearing is vital in developing language skills. Children’s language, functional performance, speech perception, and psychosocial skills showed more significant improvements if the intervention started earlier for both CI and hearing aids. This was true regardless of age, with the benefits being greater for those with worse pre-intervention hearing [28]. Moreover, the presence of additional disabilities was significantly associated with worse language outcomes for children using CIs. In children using hearing aids with additional disabilities, earlier fitting led to better language outcomes, less hearing loss, higher cognitive ability, and greater use of speech for communication [28,29]. This is particularly relevant for USH since patients usually develop retinitis pigmentosa around adolescence. Overall, it is clear that earlier intervention leads to better outcomes, particularly if the patient has multiple disabilities, as is the case with USH. 

### 1.5. Current Management

Management of USH focuses on maximizing hearing prior to the onset of retinitis pigmentosa. Hearing aids are frequently used in USH patients and are often the preferred method of management initially. However, hearing aids may be ineffective for USH1 and some USH2 and USH3 patients [30]. Therefore, CI is the appropriate option in the management of hearing loss for many patients with USH. CI enhances speech intelligibility and improves the overall quality of life in patients with USH [14,31,32]. Auditory rehabilitation after CI further enhances communication skills in these patients. 

Children with USH1 have profound hearing loss at birth and are considered prelingually deaf. Despite this, hearing in the lower frequencies is typically preserved [6,21]. These children are, therefore, the ideal candidates for CI. Early and bilateral CI leads to better audiological performance in USH1 patients [1,9,31,33,34]. Auditory rehabilitation for USH2 patients typically starts in early childhood with the use of hearing aids. In USH2 patients with severe progressive hearing loss, poor speech discrimination and communication difficulties may persist even with hearing aids [32]. In these cases, CI is the next step in auditory rehabilitation. In USH3 patients, hearing loss can be pre-lingual or post-lingual but typically begins mainly before the third decade of life [14]. In these instances, cochlear implants are also the appropriate option for USH3 patients [35].

Although early CI has been proven to improve outcomes in certain USH patients, particularly congenitally deaf USH1 patients, there is limited information on the most beneficial timing for cochlear implantation for patients who currently have residual hearing and a progressive form of the disease. Interestingly, studies have shown that only 10% of eligible USH patients have received CI. Moreover, USH is often diagnosed late, and implantation occurs even later, with an average age at implantation of 5.4 years (age range of the participants 2–15 years). The objective of this study is to perform a systematic review to evaluate the audiological performance of USH patients after CI to better understand who would benefit from CI and the ideal time for implantation. We hope that this will lead to better hearing and speech outcomes for patients while also reducing the cost burden on patients prone to less favorable outcomes.

## 2. Materials and Methods

### 2.1. Search Strategy

A protocol of this systematic review was designed a priori and was registered in the PROSPERO database (registration number is CRD42020204537). Following the Preferred Reporting Items for Systematic Reviews and Meta-analysis (PRISMA) checklist, studies were compiled and screened by two independent reviewers using the software Covidence. PubMed, Embase, Cochrane library, and CINAHL electronic databases were searched. There were no search restrictions based on year, language, or publication type; however, the CINAHL search was restricted to academic journals. Searches were performed using keywords such as Usher Syndrome, Graefe-Usher, retinitis pigmentosa deafness syndrome, cochlear implants, and auditory prostheses. Due to the limited trials and reviews available on USH, only the term “Usher Syndrome” was searched in the Cochrane library. Searches were last performed on 10 November 2019. Before de-duplication and screening, these searches yielded 69 results from PubMed, 112 from Embase, 16 from the Cochrane library, and 27 from CINAHL. The specific searches performed are displayed in Table 2. 

### 2.2. Eligibility of Studies

Study eligibility was determined using the PICOS (Population, Intervention, Comparators, Outcome measure, and Study design) framework. Study inclusion criteria were defined as: Usher Syndrome patients regardless of age, nationality, or clinical subtype who underwent CI, post-implantation auditory, cognitive, and lifestyle outcomes in comparison to pre-implantation values, and any clinical study design excluding reviews. 

**Figure 1:**
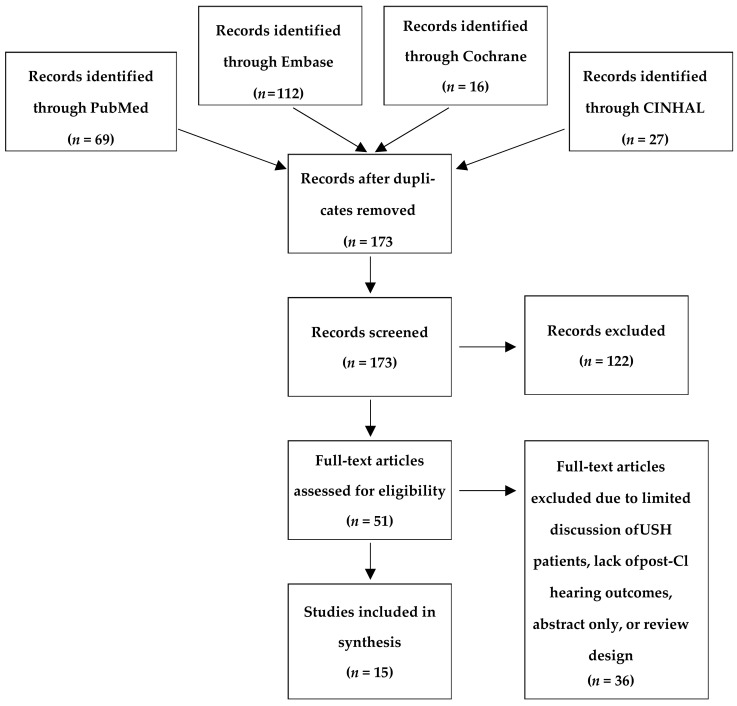
The PRISMA flowchart diagram for literature searching and screening.

When investigating the optimal time for CI in USH management, 224 studies were found in an online database search. After screening all abstracts for relevance and removing duplicates, 51 studies remained for inclusion in this study. Next, 36 studies were then excluded after full-text review based on the inclusion criteria above, leaving 15 studies for inclusion in this systematic review. Most studies excluded at this stage were due to a lack of information specific to USH patients or incompatible study design. Two papers were excluded because no full English translation was available. The study inclusion criteria are outlined in Figure 1.

## 3. Results

### 3.1. Multi-Patient Studies

Ten multi-patient papers met inclusion criteria and are summarized in Table 3. Of these papers, no study directly addressed the optimal hearing level at which to consider USH patients for cochlear implantation. However, all papers demonstrated that CI in this population was beneficial in several domains. Improvements in hearing, cognitive, and quality of life outcomes were reported post-implantation. When considering auditory outcomes, a case-control study included nine USH patients and controls matched for the age of implantation and duration of device use. It was observed that USH patients outperformed their matched CI controls with respect to mean pure tone average post-operatively. These USH patients also had excellent outcomes in speech intelligibility and auditory abilities [36]. However, this study failed to include the specific subtype and mutations of USH, limiting the interpretation of their results. Additionally, three of the nine USH patients were siblings from a highly motivated family, which may skew the results towards more positive outcomes. Another study, including 26 patients, found that USH patients implanted bilaterally at younger ages had more favorable outcomes achieving oral communication skills [1]. In contrast, those implanted at later ages, after longer durations of deafness, scored sub-optimally in speech reception [37]. Additionally, only 3 of 26 patients opted in for genetic testing, all of whom were positive for the MYO7A mutation. Although this is informative, a complete mutation analysis would likely provide more informative results on the utility of CI in USH patients.

Early CI in USH patients is also supported when analyzing nonauditory outcomes. USH1 children implanted before the age of two years have similar phonological memory to normal-hearing children and children who used hearing aids. However, children implanted after the age of two had outcomes similar to other deaf children with CI [38]. Meanwhile, a study demonstrated that CI in USH1 adults led to an average equivalent hearing loss of 107.1 dB HL, while CI in USH1 children resulted in an equivalent hearing loss of 84.4 dB HL [34]. In contrast, the non-CI users were all profoundly deaf with pure-tone average scores above 110 dB at 0.5, 1, and 2 kHz. In addition, significant improvement in quality of life of implanted individuals was observed as measured by the Nijmegen Cochlear Implant Questionnaire (NCIQ), with increases in an equivalent hearing loss correlating to decreased sound perception measures on the NCIQ [34].

Baseline hearing and speech abilities of patients, prior to implantation, as well as the benefits of early implantation were examined in six papers included in this study [7,31,32,35,39,40]. A majority of these patients had profound baseline pre-lingual deafness. USH1 patients implanted within the first two decades of life experienced improved quantitative auditory outcomes with CI [7,31]. The limited genetic analysis revealed the only patient to report adverse outcomes had a CDH23 mutation [7]. However, no conclusions can be confidently drawn since this is only one patient. Moreover, a study observed in a group of 10 patients with USH1, implanted on average at 18.5 years, that one year after CI implantation, only three patients improved their sentence recognition by 40%, 30%, and 10% respectively. Sound detection was achieved by the majority of patients in this study and showed that even late implantation could provide access to sound [39].

**Table 3:** Summary of findings from multi-patient studies.

References	USH Subtype	Pre-CI Hearing Measurement	Average Age at CI (Years)	Average Duration of Implant Use at Time of Audiological Evaluation (Years)	Post-CI Hearing Measurement	Other Findings
Alzhrani et al. [36], 2018	Not Mentioned (*n* = 9)	Not mentioned	5.3 (3–7.6)	3	Measurement 3 years post CI. All scored the maximum of five on the SIR, and most of them had a CAP score of 9. Only one subject failed to exceed a CAP score of 5, although he haEHLd a very high SIR score of 5 Average PTA = 23.67	
Broomfield et al. [37], 2013	Not Mentioned [37], (congentially deaf from birth, likely USH1) (*n* = 9)	[37] Not Mentioned	Early = 2.7 Late = 12.73	10.3 (4.8–11.5)	Early: SRS = 5.5 Late: SRS = 4	Outcomes are often excellent but can be variable even within the same syndrome groups
Damen et al. [34], 2016	USH1 (*n* = 14)	>110 dB at 0.5, 1, and 2 kHz	Children = 12.4 ± 2.9 Adults = 30.7 ± 6.8	9.2 (3.0–15.7)	CI in USH1 children resulted in EHL of 84.4 dB HL	
Hartel et al. [32], 2017	USH2 (*n* = 8)	Average phoneme score = 41%	59	4.4 (1–19)	Average phoneme score = 87%	Postoperative quality of life and speech production improvements is greater in those with post-lingual deafness than those with pre-lingual deafness (USH1), as determined by NCIQ.
Hoshino et al. [39], 2017	USH1 (*n* = 10)	Average PTA HA = 103 dB HL	18.9 (5–49)	11.4 (1–27)	Average PTA CI = 35 dB	3 patients improved sentence recognition and 5 patients were able to improve in speech detection. Late implantation limits speech perception
Jatana et al. [1], 2013	USH1 (*n* = 3)	Not mentioned.	Mean age of CI for patients born before 1992 * = 4.3 (3.3–7.1)	7.8 (1–15.6)	92.3% were able to achieve some level of open-set speech perception on age-appropriate testing	All but 2 children in the current series were able to develop some open-set speech discrimination, and 69.2% were using oral or primarily oral communication by time of last follow-up.
	USH unspecified (*n* = 23)		Mean age of CI for patients born after 1992 * = 1 (0.5–11.6)			
Liu et al. [7], 2008	USH1 (*n* = 9)	Hearing threshold = 110 dB	5.4 years (2–11)	1.5 (1–2)	Hearing threshold at 0.5 kHZ = 46	All patients showed post-implantation improvements.
Loundon et al. [40], 2003	USH1 (*n* = 11)	Closed set perception scores = 0%	9.3 (1.5–44)	4.4 (0.8–9)	Closed set perception score = 84% (mean)	Although all patients perceived no closed or open set words prior to implantation, children implanted before 9 years of age had the best perceptive results.
	USH3 (*n* = 3)					
	Unspecified (*n* = 1)	Open set perception scores = 0%				
Pennings et al. [31], 2006	USH1 (*n* = 14)	Not mentioned.	10 (3.5–30.4)	11.4 (2–28)	Significant reduction in EHL in 5 of 7 patients implanted before age 10, with a mean EHL of 84 dB HL. The mean EHL increased for those implanted at later ages.	Cochlear implantation within the first two decades of life results in improvement in 93% of patients, all of whom had profound hearing loss at baseline.
Pietola et al. [35], 2012	USH3 (*n* = 19)	Hearing threshold = 110 ± 8 dB HL (PTA = 0.5–4 kHz) and Hearing aid threshold = 58 ± 11 dB HL	41 ± 17	1 (0.5–1.5)	Hearing threshold = 34 ± 9 dB HL	All patients used hearing aids preoperatively, and all benefited from CI as evidenced by improvements in PTA post-CI and the Glasglow Benefit Hearing and speech discrimination, age at implantation and the change in the hearing ability varied significantly after implantation.

USH: Usher syndrome; CI: Cochlear implantation; CAP: Central Auditory Processing; SIR: Speech Intelligibility Rating; SRS: Speech recognition scores; PTA: pure tone audiometry, measured in decibels hearing level; NCIQ: Nijmegen Cochlear Implant Questionnaire; EHL: Equivalent hearing loss; * 1992 was the year universal newborn hearing was mandated.

Similarly, a study reported improved communication abilities in children of multiple USH subtypes implanted before age nine [40]. Mutation analysis of five of the study patients found that only three patients carried the USH1 mutations. This speaks to the heterogeneity of USH1 but could also be due to the presence of previously unmapped loci or experimental error. Another study showed that USH1 patients implanted before age three had the best post-operative speech perception results in the study sample, further supporting CI at younger ages [39].

USH2 and USH3 patients who used hearing aids pre-operatively also experienced measurable benefits from CI with respect to pure tone audiometry, speech perception, and quality of life regardless of age at implantation [32,35]. They found that the average preoperative PTA was 110 ± 8 dB with an average aided hearing level of 58 ± 11 dB. The post-operative hearing level improved to 34 ± 9 dB and word recognition scores were significantly better as well. Critically, they also found no correlation between the age of implantation and the Glasgow Benefit Inventory (GBI) for CI in USH3. Much like the clinical presentation, post CI outcomes were variable despite the homogenous genetic background [35].

Overall, the multi-patient studies suffered from a few limitations. First, many prospective studies, such as Loudon et al., had relatively small sample sizes, <10 patients. While this is expected given the disease’s rarity, it presents challenges to their conclusions’ external validity. It is also important to note that retrospective, cross-sectional studies are also potentially limited by difficulty in making causal inferences and sampling bias, particularly with rare diseases. Finally, drawing associations between specific phenotypes and post-CI clinical outcomes is limited by lack of consistent genetic testing of patients.

### 3.2. Case Reports

We found four relevant case reports that discussed the outcomes of CI in USH patients of different ages. In 2015, a study reported audiological outcomes of a 5-month-old USH1 patient born with profound bilateral deafness who underwent simultaneous bilateral CI at five months of age [33]. Six months post-operatively, hearing thresholds were 15 dB HL at 250 Hz and 25 dB HL at 500–8000 Hz. After eight months, the child’s audiological development was age-appropriate, scoring above the norm-curve on the LittlEars Auditory Questionnaire, achieving a five on the Categories of Auditory Performance test and producing up to 10 functional words [33]. Interestingly, this study did not address any of its limitations. Notably, it omitted the patient’s subset of USH, genetics, and preoperative audiologic data, limiting the conclusions that can be drawn from post-operative outcomes. However, the results of this study can be useful in providing preliminary support for positive outcomes after bilateral CI in children under 12 months old. 

In another study, there are also several cases of USH patients with post-lingual deafness who underwent CI. An USH2 patient who used hearing aids since age 2 was deaf in the left ear and had open-set sentence recognition in the right ear eight months after right CI at 34 years old. She then underwent left ear CI. One to two years after sequential bilateral CI, the patient had pure tone audiometry (PTA) of 26 dB HL with left CI and 35 dB HL with the right one. Everyday sentence scores were 20% and 100% at 65 dB, respectively [41]. Another USH patient, subtype not specified, was implanted at age 52 after rapidly reaching profound deafness in her second and third decade of life. The patient achieved PTA of 30–35 dB HL after CI while pre-op values were 105–115 dB HL. Importantly, the patient used hearing aids until age 29, after which no benefit was observed [42]. Finally, another study reported the ability of an implanted USH3 patient to understand daily conversation after CI. It was observed that patient was able to recognize 100% of vowels and 52% of consonants on recognition tests after implantation at 35 years of age [43]. This patient also has enhanced cortical activation in both primary auditory and auditory association areas post-operatively, as observed by positron emission tomography (PET) [43]. Importantly, as with the first case study, none of these cases reported preoperative audiological data, genetic mutation data and omitted the precise type of USH. While this may not have affected the studies’ original objective, it limits the utility in determining CI outcomes at different ages and in the various subsets of USH. A summary of the different characteristics of each case report discussed is shown in Table 4.

## 4. Discussion

CI is known to have a significant role in the management of progressive hearing loss [44]. CI before two years of age, regardless of the cause of hearing loss, has been shown to produce age-appropriate lexical skills as well as receptive and expressive language outcomes that persist until at least mid-elementary school age [45]. Patients who undergo CI before two years old also demonstrate improved vocabulary and speech perception [45,46,47]. Early implantation’s benefits stem from increased neural plasticity at younger ages and continued auditory stimulation before significant sensory deprivation, which can alter auditory pathways in the central nervous system [48]. The earlier a patient undergoes CI, the more likely they will experience functional audiological outcomes that allow for communication with others in the setting of sensory loss.

A study explored the hypothesis that CI performance is affected by the site of cochlear pathology. It was concluded that defects of the spiral ganglion were correlated with poor CI performance while defects of the membranous labyrinth and hair cells were correlated with favorable CI performance [25]. Although this study excluded patients with syndromic hearing loss, their hypothesis was validated by another study in a follow-up investigation. It was observed that USH1 patients with the CDH23 mutation, a cell-cell adhesion protein, demonstrated unfavorable CI outcomes based on audiological evaluation [5,31]. However, they also observed favorable CI outcomes in USH1B patients with the MYO7A mutation, which leads to defects in the hair bundles [31]. This mutation accounts for about 75% of all USH1 patients and correlates with the favorable outcomes of cochlear implantation in USH1 patients [49]. More research must be done to elucidate the exact pathology of CDH23 and confirm its association with adverse CI outcomes.

Usher syndrome’s multi-sensory effects make early implantation even more critical as it is vital to maximize one’s communication skills before visual loss occurs in order to have the capacity to interact with others in a meaningful way. Based on the studies above, early implantation in USH can be defined as CI within the first decade of life but varies significantly between each study and depends on the clinical subtype of patients examined and whether patients with bilateral CI were implanted sequentially or simultaneously. Interestingly, although CI prior to 12 months of age has been avoided in the past due to anesthesia and post-operative complication risks, bilateral CI has been shown to be not only safe but also successful in improving auditory outcomes in an USH patient as young as five months of age [33]. With this possibility in mind, earlier implantation and its associated risks should also be further investigated.

Early intervention in USH is not always possible. A delayed diagnosis of USH combined with socioeconomic factors and lack of education about multi-sensory diseases may be a barrier to early implantation and potentially lead to suboptimal communication outcomes compared to age-matched controls. Auditory deprivation induces central and peripheral nervous system changes in both pre-lingual and post-lingual deafness. Studies have shown that patients with congenital deafness can develop near-normal hearing when they have auditory function restored before 3.5 years old [48]. It has been observed that in patients with USH1 who underwent CI at an average age of 18.5 years had significantly worse outcomes than younger patients one year after CI [39]. Only three patients significantly improved their speech perception performance in sentence recognition, and five patients had only sound detection. Sound detection was achieved by the majority of patients and showed that late implantation of CI could only provide access to sound [39]. This may not be enough to improve the interaction with patients’ environment and overall quality of life. It is essential to reconsider the surgical indication if a patient developed without auditory stimulation and relied solely on sign language before deciding if cochlear implantation will benefit them. In this regard, the role of a multidisciplinary approach, including psychologists, school educators, and various physicians should not be underestimated. This highlights the importance of newborn screenings combined with selective genetic testing for the early diagnosis of hearing loss and possibly USH.

Although definite conclusions are difficult to draw from the studies analyzed, a common theme is that CI was beneficial in all cases of USH given that the patient was not post-lingually deaf regardless of the age of implantation. However, it is essential to note that all of the patients implanted after age 30 were post-lingually deaf. This is a key caveat as not all USH patients fall into this category and highlights the importance of early evaluation.

The case study of the five-month-old with congenital deafness undergoing CI is particularly interesting as well. This very early age of implantation resulted in age-appropriate hearing outcomes within one year of follow-up. This case highlights the possibility of effective and safe simultaneous bilateral CI in infants with USH. This is important because simultaneous bilateral CI has been shown to produce improved speech perception when compared to unilateral CI in pediatric patients [50]. Simultaneous bilateral CI provides electrophysiological and behavioral benefits when compared to patients who undergo sequential bilateral CI over at least six months, with differences primarily attributable to the binaural squelch and head shadow effect that occurs with unilateral CI [51,52].

The multi-patient studies discussed above provide larger sample sizes from which more firm conclusions can be drawn. Overall, CI is beneficial for USH patients regardless of clinical subtype or age at implantation in multiple domains. USH patients who receive CI not only experienced improvements in hearing and communication but also in their quality of life. USH1 patients who get the most benefit from cochlear implantation are implanted early, at a very early age or after the maintenance of auditory stimulation with hearing aids [7,31,32,35,39,40]. Concerning the timing of CI, while in USH1 found that patients who are implanted at older ages still benefit from CI due to increased sound detection, their benefits decrease with age without previous auditory stimulation at older ages [39].

Regarding USH2 and USH3, patients who used hearing aids pre-operatively experienced measurable benefits from CI with respect to pure tone audiometry, speech production, and quality of life regardless of age at implantation [32,35]. Moreover, due to hearing and speech’s central role in interacting with society, these benefits extend beyond hearing alone. In a study of CI in USH3, patients had fewer physical health, mental health, and social trust problems than those without CI [53]. This lends support for more widespread CI in not only congenitally deaf patients but those with less severe forms of sensorineural hearing loss as well.

CI indications in patients with adequate residual hearing but a progressive form of the disease is much less clear. Even within the same subtype of USH and genetically homogenous communities, the progressive hearing loss can be highly variable. Optimal CI timing in patients with significant residual acoustic hearing is an ongoing debate in the literature given the advantages of acoustic function with hearing in noise when compared to electrical stimulation [54]. CI’s may not be optimal in noise, and it may sometimes be beneficial to wait and make use of acoustic hearing before deciding to implant USH patients. More studies are warranted to determine the risk of losing residual hearing following implantation and outcomes per USH genotype before recommendations can be made.

This study’s main conclusion is that the large majority of USH patients stand to benefit from CI. The main factor limiting CI’s positive outcomes for patients is the absence of previous exposure to auditory stimulation and language use for those implanted at older ages. Without these key pre-requisites, USH patients reap fewer functional benefits from CI or may be disinclined to use it. Therefore, particular attention should be paid to the patient’s clinical history before CI is considered especially in light of the high lifetime cost of CI, which is approximately >$1,000,000 [25,26].

### Limitations

The studies mentioned above are limited by small sample sizes and often incomplete descriptions of pre-implantation auditory function. Moreover, the lack of consistent genetic testing severely hampers a more detailed analysis of CI with specific genetic types of USH. Many of the studies lacked standardized assessment tools. For example, some studies did not report preoperative PTA data and others only reported language comprehension scores. Finally, our search did not include unpublished studies, non-English language, or studies not archived on the databases queried were not included in the systematic review.

## 5. Conclusions

This systematic review focused on appraising the implementation of CI in USH and analyzing who would benefit most from implantation. The pathogenesis and progression of the USH subtypes informs their treatment. In USH1, patients are born with profound deafness accompanied with vision loss within the first decade of life. In congenitally deaf patients, earlier and bilateral CI has better auditory outcomes, speech outcomes, and childhood development than later and unilateral CI. Therefore, for USH1 early, bilateral CI as soon as feasible is the best course of action. Successful bilateral implantation as early as five months has been reported. 

USH2 is characterized by congenital moderate to severe bilateral hearing loss, retinal degeneration starting as early as adolescence, while USH3 patients have a highly variable course of progressive hearing loss with variable age of onset, progressive vestibular dysfunction, and often a later onset of retinal degeneration. This variability in presentation combined with the diseases’ rarity make drawing firm conclusions difficult. While all of the studies examining USH2 and USH3 indicated that CI improved patients’ hearing and quality of life, none of them provided clear evidence concerning the optimal time of CI given the progressive nature of USH2 and USH3. Pre-implantation hearing should be amplified with hearing aids, which maintains auditory pathway stimulation and may result in better outcomes post-CI in these patients. Despite limited studies, there is a consensus in the literature that cochlear implantation in USH leads to better outcomes in pure tone audiometry, speech production, and quality of life regardless of age at implantation. Therefore, CI may be considered in USH2 and USH3 as soon as it is evident that traditional treatments such as hearing aids do not provide enough more benefit to the patient.

The largest gap in the literature appears to be an association between a patient’s genotype, clinical progression, and post-CI outcomes. Future studies should explore this association through multi-center case-controlled studies to gather a sufficient number of patients while simultaneously capturing the genetic scope of USH. In addition, further investigations are warranted to pinpoint which genotypes are amenable to earlier CI and the optimal timing of CI to minimize residual hearing loss versus the benefit of the implant. It is hoped that advances in hearing preservation using otoprotective therapies during implantation to reduce electrode insertion trauma will help in optimizing CI outcomes without losing the benefit of residual hearing [55,56,57]. It is also imperative to include standard measures of pre- and post-operative audiological outcomes to ensure comparability between previously published data.

## Figures and Tables

**Table 1:** A summary of Usher syndrome subtypes.

Clinical Subtype	Genetic Subtype	Gene-Function	Hearing Loss	Vestibular Function	Ocular Manifestations
Usher type 1	USH1A	Withdrawn	Profound congenital HL	Abnormal or absent vestibular sense	Early onset RP
	USH1B	MYO7A—Motor protein			
	USH1C	USH1C—Scaffold protein			
	USH1D	CDH23—Cell adhesion protein			
	USH1E	Unknown			
	USH1F	PCDH15—Cell to cell adhesion protein			
	USH1G	USH1G—Scaffold Protein with Ankyrin repeat and SAM Domain			
	USH1H	Unknown			
Usher type 2	USH2A	Usherin—Transmembrane protein	Prelingual onset of moderate to severe high-frequency sloping HL	Normal	Onset of RP in 2nd decade of life
	USH2B	ADGRV1—Transmembrane receptor protein			
	USH2C	VLGR1—Transmembrane receptor protein			
	USH2D	Whirlin—Scaffold Protein			
Usher type 3	USH3A	USH3A—Clarin 1 Transmembrane Protein	Variable onset of progressive HL	Variable	Variable onset

HL: Hearing Loss, RP: Retinitis pigmentosa.

**Table 2:** A Summary of Literature Searches Performed.

Database	Search
PubMed	(“Usher Syndromes”[Mesh] or usher[tw] or ushers[tw] or usher’ [tw] or Graefe-Usher[tw] or Hallgren[tw] or (Retinitis Pigmentosa Deafness Syndrome[tw]) or ((“Retinitis Pigmentosa”[Mesh:NoExp] or Retinitis Pigmentosa[tw]) AND (“Deafness”[Mesh:NoExp] or deaf*[tw])) or USH1B or USH1C or USH1D or USH1E or USH1F or USH1G or USH1H or USH2A or USH2C) AND (“Cochlear Implants”[Mesh] Or (“Cochlea”[Mesh] or “Ear, Inner”[Mesh] or auditory[tw] or cochlea[tw] or cochlear[tw] or cochleas[tw] or intracochlear[tw] or intra-cochlear[tw] or inner ear[tw] or hearing[tw]) AND (“Prostheses and Implants”[Mesh] or Prosthes*[tw] or prosthetic[tw] or Implant[tw] or Impants[tw] or Implantation[tw] or Implanted[tw] or Device[tw] or Devices[tw] or artificial[tw])
Embase	(‘cochlea prosthesis’/exp OR ‘cochlea prosthesis’:ti,ab OR ‘nucleus hybrid l24’:tn,ti,ab OR ‘artificial cochlea implant’:tn,ti,ab OR ‘auditory prostheses’:tn,ti,ab OR ‘cochlea implant’:tn,ti,ab OR ‘cochlea prosthesis’:tn,ti,ab OR ‘cochlear implant’:tn,ti,ab OR ‘cochlear implants’:tn,ti,ab OR ‘cochlear prostheses’:tn,ti,ab OR ‘cochlear prosthesis’:tn,ti,ab OR ‘hearing prosthesis’:tn,ti,ab OR ‘prosthesis, cochlea’:tn,ti,ab) AND (‘usher syndrome’/de OR ‘usher’ OR ‘ushers’ OR ‘usher$s’ OR ‘graefe-usher’ OR hallgren)
CINAHL	((usher’s syndrome or usher syndrome or usher) AND (cochlear implant or cochlear implants or cochlear implantation)) OR ((MH “Usher’s Syndrome”) AND (MH “Cochlear Implant”)) OR ((MH “Usher’s Syndrome”) AND (MH “Prostheses and Implants”)) OR (AB usher syndrome AND AB cochlear implant))

**Table 4:** Features of case reports.

References	USH Subtype	Pre-Operative Hearing	Age at CI	Post-Operative Outcome Measures
Alsanosi [33], 2015	Unspecified	Congenital profound bilateral deafness	5 months	PTA (25 dB HL at most frequencies), questionnaires, and CAP testing 6–8 months post-op
Derinsu & Ciprut [42], 2002	Unspecified	Post-lingual profound deafness	52 years	PTA (30–35 dB HL across most frequencies) and serial speech perception tests 6 months-4 years post-op
Ruiz & Garcia [41], 2013	USH2	Post-lingual deafness with CI in R ear 8 months prior	34 years	PTA (26 and 35 dB HL in left and right, respectively) at 16–25 months post-op
Shiomi et al. [43], 1997	USH3	Post-lingual deafness	35 years	Vowel and consonant identification and cortical activation 3 months post-op

USH: Usher syndrome; CI: Cochlear implantation; PTA: pure tone audiometry, measured in decibels hearing level; CAP: central auditory processing.

## Data Availability

No new data were created or analyzed in this study. Data sharing is not applicable to this article.
